# An Improved and Practical Method for Synthesizing of α-Sanshools and Spilanthol

**DOI:** 10.3389/fchem.2020.00187

**Published:** 2020-03-17

**Authors:** Akira Nakamura, Kazuki Mimaki, Ken-ichi Tanigami, Tomohiro Maegawa

**Affiliations:** School of Pharmaceutical Sciences, Kindai University, Osaka, Japan

**Keywords:** sanshool, stereoselective synthesis, Wittig reaction, polyene, natural products

## Abstract

An efficient and practical route for the synthesis of α-sanshools and spilanthol is described herein. Several modifications of an existing method enabled the preparation of the (2*E*,6*Z*,8*E*,10*E*)-tetraene precursor of hydroxy-α-sanshool in good yield. A highly selective Wittig reaction employing newly synthesized phosphonium salt with low deliquescence and long-term stability yielded the desired *Z*-form tetraene. This improved methodology was shown to be applicable to the efficient synthesis of α-sanshool and spilanthol.

## Introduction

**Figure 1:**
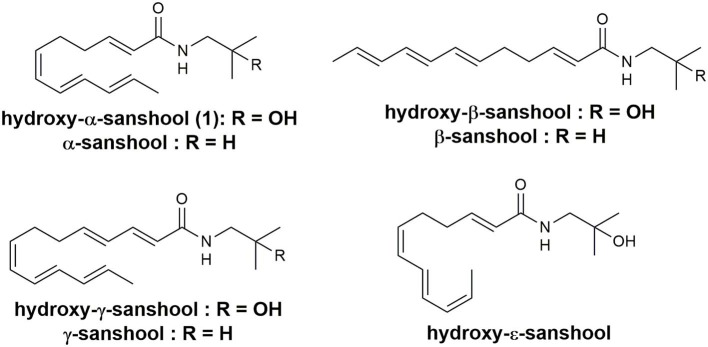
Sanshool compounds.

Sanshools are a family of polyunsaturated fatty acid amides, differing in the length and double bond geometry of the polyunsaturated (Figure 1), found in various *Zanthoxylum* species (Jang et al., 2008; Devkota et al., 2012; Greger, 2016). The various biological activities of hydroxy-α-sanshool **1** have attracted a great deal of interest in the scientific community (Koo et al., 2007; Bautista et al., 2008; Yang, 2008; Munekage et al., 2013; Tang et al., 2014; Kubota et al., 2015). However, the inherent instability of their conjugated (6*Z*,8*E*,10*E*)-triene structures, which are prone to isomerization, oxidation, polymerization, and/or photo-degradation, make sanshools difficult to isolate from natural products (Yang, 2008).

The synthesis of **1** has been reported previously by two independent research groups. Igarashi and co-workers developed two stereoselective approaches to hydroxyl-α-sanshool synthesis, both employing several metal reagents and requiring precise operations (Aoki et al., 2012; Igarashi et al., 2012). Toy and co-workers constructed a (6*Z*,8*E*,10*E*)-conjugated triene precursor moiety with moderate selectivity (6*Z*:6*E* = 2:1) using the Wittig reaction; a pure stereoisomer was isolated by recrystallization (Wu et al., 2012). The purpose of the current study was to produce high-purity hydroxy-α-sanshool **1**. Among the three existing synthesis methods, Toy's is the simplest due to the use of more conventional reagents and procedures. Our synthesis of **1** via Toy's method, however, proved difficult when following the literature, and resulted in reduced yields due to the instability or deliquescence of intermediate species. Therefore, we set out to enhance the general practicality and robustness of Toy's method of sanshool synthesis.

## Results and Discussion

**Scheme 1:**
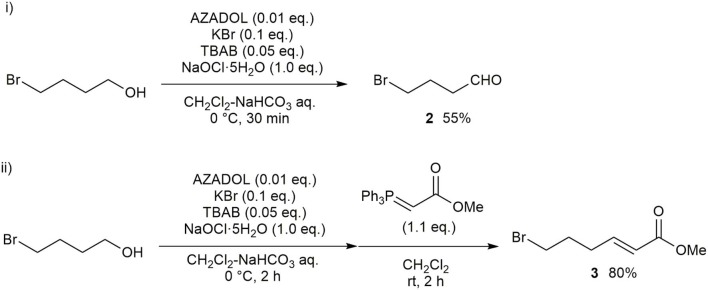
Synthesis of ester **3**.

Our synthesis of hydroxy-α-sanshool began with the oxidation of 4-bromobutan-1-ol with PCC, which was poorly reproducible on the gram scale. A more effective strategy was catalytic oxidation using commercially available AZADOL as the catalyst and sodium hypochlorite pentahydrate (NaClO·5H_2_O) as a co-oxidant (Scheme 1) (Okada et al., 2014). The desired 4-bromobutanal **2** was produced in 55% yield together with small amounts of 4-bromobutanoic acid. These results were reproducible even on the gram scale (Scheme 1, i). Other nitroxyl radical catalysts did not improve the yield of **2**. Note that partial decomposition of **2** during purification resulting in moderate overall yields. Then, the Horner–Wadsworth–Emmons (HWE) reaction was conducted, resulting in ester **3** in 80% yield (Scheme 1, ii).

**Scheme 2:**
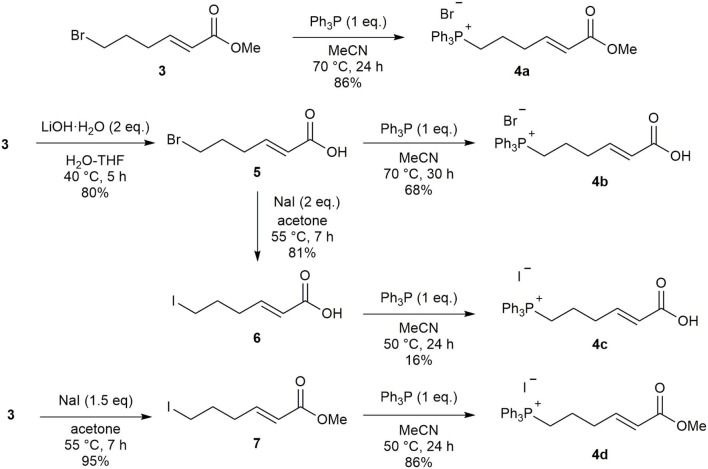
Investigation of phosphonium salt.

In an effort to improve the selectivity of the Wittig reaction (6*Z*:6*E* = 2:1), we converted ester **3** to its corresponding phosphonium salt **4a** with PPh_3_ according to Toy's synthesis method. However, this reaction suffered from low reproducibility due to the high deliquescence of **4a**. We therefore evaluated several methods to create a phosphonium salt **4** with lower hygroscopicity (Scheme 2). First, ester **3** was hydrolyzed to carboxylic acid **5** and the phosphonium salt **4b** was obtained in good yield by the reaction with PPh_3_. Unfortunately, **4b** exhibited deliquescence similar to that of **4a**. To determine the influence of the phosphonium salt counter anion on deliquescence, we prepared the iodonium salt **4c** using the corresponding alkyl iodide **6**. However, this also resulted in a compound with high deliquescence. We found that the combination of counter anion and functional group is important in determining the deliquescence of phosphonium salts, and obtained the non-deliquescent iodine salt **4d** from the iodo ester **7**.

We next examined the stereoselective synthesis of tetraene **8** with **4d**. The results of the Wittig reaction of (2*E*,4*E*)-2,4-hexadienal **9** with phosphonium salt **4d** under various reaction conditions are summarized in Table 1. When *t*-BuOK or NaH was used as a base, tetraene **8** was obtained in moderate yields and 6*Z*/6*E* stereoselectivity (entries 1 and 2). The use of potassium bis(trimethylsilyl)amide (KHMDS) as a base afforded the best results. The use of KHMDS at −40°C improved the stereoselectivity of the product to 12:1, but with a slight decrease in yield (Entry 3). Conducting the reaction at −78°C failed to yield the desired product **8** (Entry 4). However, gradually increasing the temperature to −40°C from −78°C, after the addition of **9** to the ylide generated from **4d**, resulted in tetraene **8** in high yield and high 6*Z*/6*E* selectivity (Entry 5) (Uchiyama et al., 2017). However, other tetraene isomers, derived from small amounts of stereoisomers contained in commercially available (2*E*,4*E*)-2,4-hexadienal **9**, were still observed. We finally succeeded in obtaining (2*E*,6*Z*,8*E*,10*E*)-tetraene **8** as a single isomer in 83% yield by using pure (2*E*,4*E*)-2,4-hexadienal **9** prepared from (2*E*,4*E*)-2,4-hexadien-1-ol with manganese oxide.

**Table 1:** Optimization of Wittig reaction.

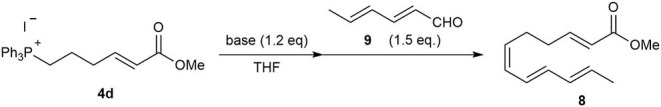				
**Entry**	**Base**	**Conditions**	**Yield (%)**	**6*Z*:6*E*^a^**
1	*t*-BuOK	0°C, 12 h	52	5:1
2	NaH	rt, 3 h	60	4.5:1
3	KHMDS	−40°C, 2 h	43	12:1
4	KHMDS	−78°C, 6 h	–	–
5	KHMDS	−78°C, 0.5 h to −40°C, 2 h	83	>20:1

a *The ratio of stereoisomers was determined by ^1^H NMR analysis*.

**Scheme 3:**
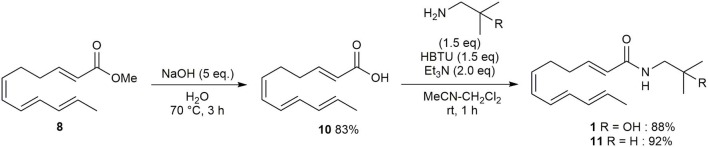
Synthesis of hydroxy-α-sanshool **1** and α-sanshool **11**.

Then, following Toy's method, ester **8** was converted to carboxylic acid **10** in 83% yield (Scheme 3). Amide formation, via the coupling of **10** and the appropriate amine using HBTU and Et_3_N, afforded hydroxy-α-sanshool **1** and α-sanshool **11** in 88% and 92% yields, respectively (please see Supplementary Material).

**Scheme 4:**
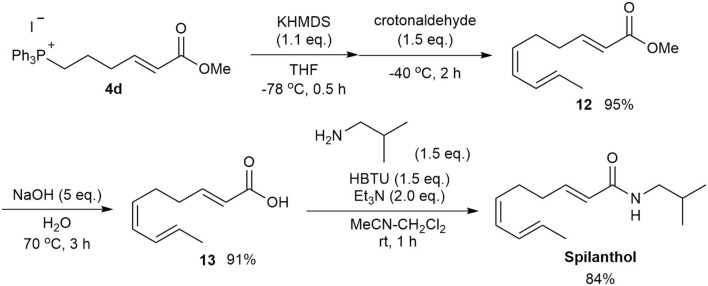
Synthesis of spilanthol.

The developed method was applied to the synthesis of the biologically active compound spilanthol (Sharma et al., 2011; Barbosa et al., 2016), also known as affinin, which contains a (2*E*,6*Z*,8*E*)-decatrienamide moiety (Scheme 4). Several synthetic methods for spilanthol have been reported (Crombie et al., 1963; Ikeda et al., 1984, Ikeda et al., 1987). A recent short step synthesis by Pastre provided high stereoselectivity, but suffered from a relatively low overall yield of 18% (Alonso et al., 2018). Our synthesis, starting from the Wittig reaction of the ylide generated from **4d** and crotonaldehyde to afford ester **12**, resulted in a 95% yield of the (2*E*,6*Z*,8*E*)-single stereoisomer. Saponification of **12** gave carboxylic acid **13** in 91% yield. Spilanthol was then synthesized in 84% yield using the coupling reaction employed in the α-sanshool synthesis. Thus, the efficient and stereoselective synthesis of spilanthol was achieved from 4-bromobutanol in six steps with an overall yield of 47%.

## Conclusion

We developed a practical and reproducible method for the synthesis of hydroxy-α-sanshool and α-sanshool. Notably, modifications of the Wittig reaction using a newly synthesized, non-deliquescent phosphonium salt under low-temperature conditions succeeded in forming single stereoisomers of (2*E*,6*Z*,8*E*,10*E*)-tetraene and (2*E*,6*Z*,8*E*)-triene moieties in good yields. This method was shown to be applicable to the synthesis of spilanthol in six steps, resulting in an overall yield of 47%. Further studies on the synthesis of other sanshool derivatives are ongoing.

## Data Availability Statement

All datasets generated for this study are included in the article/Supplementary Material.

## Author Contributions

AN, KM, and KT performed the experiments. AN and TM wrote the manuscript. All authors designed the experiments and were involved in the data analysis. All authors designed the experiments, were involved in the data analysis, and have expressed approval of the final version of the manuscript.

### Conflict of Interest

The authors declare that the research was conducted in the absence of any commercial or financial relationships that could be construed as a potential conflict of interest.
