# Supplementary material for: An Improved and Practical Method for Synthesizing of α-Sanshools and Spilanthol
Source: Front Chem. 2020 Mar 17;8:187. doi: 10.3389/fchem.2020.00187 (PMC7090093; doi:10.3389/fchem.2020.00187)
Supplement: Supplementary file 1 [file Data_Sheet_1.docx]

Supplementary Material

An Improved and Practical Method for Synthesizing of α-Sanshools and Spilanthol

Akira Nakamura^1^, Kazuki Mimaki^1^, Ken-ichi Tanigami^1^, Tomohiro Maegawa^1*^

^1^ School of Pharmaceutical Sciences, Kindai University, 3-4-1 Kowakae, Higashi-osaka, Osaka 577-8502, Japan.

# EXPERIMENTAL SECTION

All chemicals were obtained from Sigma Aldrich, TCI, Nakarai Chemical or FUJIFILM Wako chemical as reagent grade and were used as received. Column chromatography and TLC were performed on Merck Silica gel 60 (230‒400 mesh) and Merck Silica gel F254 plates (0.25 mm), respectively. ^1^H and ^13^C NMR spectra were recorded on the JEOL JMN-400 spectrometer in CDCl_3_. Chemical shifts (δ) are reported in ppm downfield from the internal standard tetramethylsilane (TMS). High-resolution electrospray ionization mass spectrometry (HRESIMS) was measured by Exactive Plus mass spectrometer (Thermo Fisher Scientific Inc.).

**Methyl (*E*)-6-bromohex-2-enoate (3)^1^**

To a solution of 4-bromobutan-1-ol (2.14 g, 14.0 mmol) and AZADOL (21.5 mg, 0.140 mmol) in CH_2_Cl_2_ (35 mL), saturated NaHCO_3_ aq. (15 mL) containing KBr (167 mg, 1.40 mmol) and *n*-Bu_4_NBr (226 mg, 0.701 mmol) was added. While the reaction mixture was vigorously stirred at 0 °C, a premixed solution of aqueous NaOCl･5H_2_O (2.30 g, 14.0 mmol) and saturated NaHCO_3_ aq. (20 mL) was added dropwise over 10 min. After stirring for 2 h at 0 °C, the reaction mixture was separated, and the aqueous layer was extracted with AcOEt. The combined organic layers were washed with brine, dried over Na_2_SO_4_, and partially concentrated under reduced pressure. To the concentrated solution methyl (triphenylphosphoranylidene)acetate (5.15 g, 15.4 mmol) was added and stirred at room temperature for 2 h. The solution was concentrated under reduced pressure, and purified by silica gel column chromatography (hexane/AcOEt = 5:1) to give **3** (2.32 g, 80%) as colorless oil. ^1^H NMR (CDCl_3_) δ 1.99-2.06 (m, 2H), 2.36-2.42 (m, 2H), 3.42 (t, *J* = 6.6 Hz, 2H), 3.74 (s, 3H), 5.89 (dt, *J* = 15.6, 1.6 Hz, 1H), 6.93 (dt, *J* = 15.2, 6.8 Hz, 1H).

**Methyl (*E*)-6-iodohex-2-enoate (7)^2^**

To a solution of **3** (2.30 g, 11.1 mmol) in acetone (23 mL) was added NaI (2.50 g, 16.7 mmol). After stirring at 55 °C for 7 h, the reaction mixture was quenched with saturated NH_4_Cl aq. and then extracted with AcOEt. The organic layer was dried over Na_2_SO_4_ and concentrated under reduced pressure to give **7** (2.68 g, 95%) as colorless oil that was used without further purification. ^1^H NMR (CDCl_3_) δ 1.99 (m, 2H), 2.35 (m, 2H), 3.20 (t, *J* = 6.6 Hz, 2H), 3.72 (s, 3H), 5.90 (dt, *J* = 15.6, 1.5 Hz, 1H), 6.92 (dt, *J* = 15.6, 7.2 Hz, 1H).

**(*E*)-(6-Methoxy-6-oxohex-4-en-1-yl)triphenylphosphonium iodide (4d)**

A mixture of **7** (730 mg, 2.87 mmol) and PPh_3_ (791 mg, 3.02 mmol) in acetonitrile (3 mL) was heated at 50 °C for 24 h. The solvent was removed under reduced pressure and the crude product was triturated with AcOEt and stirred for 15 min. The insoluble white powder was filtered, washed with AcOEt, and dried in vacuo to give **4d** (1.28 g, 86%) as white solid. mp 223-224 ^o^C. ^1^H NMR (CDCl_3_) δ 1.75-1.77 (m, 2H), 2.65-2.67 (m, 2H), 3.63 (s, 3H), 3.74-3.81 (m, 2H), 5.79-5.84 (d, *J* = 7.8 Hz, 1H), 6.73-6.81 (m, 1H), 7.65-7.69 (5H, m), 7.73-7.81 (m, 10H). ^13^C NMR (CDCl_3_) δ 21.2 (d, *J* = 4.2 Hz) 22.4 (d, *J* = 51.1 Hz), 32.1 (d, *J* = 17.3 Hz), 51.4, 117.8 (d, *J* = 86.5 Hz), 122.6, 130.5 (d, *J* = 12.4 Hz), 133.6 (d, *J* = 10.7 Hz), 135.1 (d, *J* = 3.3 Hz), 146.5, 166.7. HRMS (ESI+) m/z calcd for C_23_H_27_O_2_NaP [M+Na]^+^: 389.1665, found: 389.1652.

**Methyl (2*E*,6*Z*,8*E*,10*E*)-dodeca-2,6,8,10-tetraenoate (8)^1^**

A mixture of **4d** (103 mg, 0.200 mmol) in dry THF (1.0 mL) was cool to -78 °C under an argon atmosphere. KHMDS (0.48 mL of 0.5 M toluene solution, 0.240 mmol) was added dropwise to the solution over 2 min. After stirring at -78 °C for 0.5 h, (2*E*,4*E*)-2,4-hexadienal **9** (44.2 µL, 0.300 mmol) was added. After stirring for 2 h at -40 °C, the reaction mixture was quenched with saturated NH_4_Cl aq. and then extracted with AcOEt. The organic layer was dried over Na_2_SO_4_ and concentrated under reduced pressure. The residue was purified by silica gel chromatography (hexane/AcOEt = 1:1) and (hexane/AcOEt = 10:1) to give **8** (34.2 mg, 83%) as yellow oil. ^1^H NMR (CDCl_3_) δ 1.78 (d, *J* = 6.8 Hz, 3H), 2.27-2.38 (m, 4H), 3.72 (s, 3H), 5.32-5.43 (m, 1H), 5.69-5.77 (m, 1H), 5.85 (d, *J* = 16.0 Hz, 1H), 6.00-6.21 (m, 3H), 6.29-6.55 (m, 1H), 6.98 (dt, *J* = 15.6, 6.8 Hz, 1H).

**(2*E*,6*Z*,8*E*,10*E*)-Dodeca-2,6,8,10-tetraenoic acid (10)^1^**

A mixture of **8** (150 mg, 0.720 mmol) and NaOH (144 mg, 3.60 mmol) in water (1.4 mL) was stirred at 70 °C for 3 h. After cooling to room temperature, the reaction mixture was extracted with AcOEt, and then acidified with aqueous 1N HCl to pH = 2. The organic layer was washed with brine, dried with Na_2_SO_4_ and concentrated under reduced pressure to afford **10** (115 mg, 83%) as white solid. ^1^H NMR (CDCl_3_) δ 1.76 (d, *J* = 7.2 Hz, 3H), 2.30-2.36 (m, 4H), 5.30-5.36 (m, 1H), 5.71 (dq, *J* = 14.4, 6.8 Hz, 1H), 5.83 (d, *J* = 15.6 Hz, 1H), 6.00-6.20 (m, 3H), 6.29-6.35 (dd, *J* = 14.0, 11.2 Hz, 1H), 7.06 (dt, *J* = 15.6, 6.8 Hz, 1H).

**Hydroxyl-α-sanshool (1)^1^**

To a mixture of **10** (28.8 g, 0.150 mmol), 1-amino-2-methyl-2-propanol (21 μl, 0.225 mmol) and triethylamine (0.6 mL, 4 mmol) in MeCN (0.3 mL) and CH_2_Cl_2_ (0.15 mL), was added HBTU (114 mg, 0.300 mmol). After 1 h, the reaction mixture was diluted with AcOEt, and washed with 1N HCl, saturated NaHCO_3_ aq., and brine. The organic layer was dried over Na_2_SO_4_ and concentrated under reduced pressure. The residue was purified by silica gel chromatography (AcOEt) to afford **1** (35.7 mg, 88%) as a colorless oil. ^1^H NMR (CDCl_3_) δ 1.21 (s, 6H), 1.76 (d, *J* = 6.8 Hz, 3H), 2.25-2.34 (m, 4H), 2.78 (s, 1H), 3.31 (d, *J* = 6.0 Hz, 2H), 5.32-5.38 (m, 1H), 5.71 (dq, *J* = 14.4, 6.8 Hz, 1H), 5.82 (d, *J* = 15.2 Hz, 1H), 5.88 (brs, 1H), 6.00 (dd, *J* = 11.2, 11.2 Hz, 1H), 6.06-6.19 (m, 2H), 6.27-6.34 (m, 1H), 6.84 (dt, *J* = 16.0, 6.0 Hz, 1H). ^13^C NMR (CD_3_OD) 18.4, 27.2, 27.6, 33.1, 51.1, 71.6, 125.1, 126.5, 130.4, 130.6, 130.9, 133.2, 134.7, 145.1, 169.0. HRMS (ESI+) m/z calcd for C_16_H_25_NO_2_Na [M+Na]^+^: 286.1783, found: 286.1778.

**α-Sanshool (11)^3^**

To a mixture of **10** (28.8 mg, 0.150 mmol), isobutylamine (23 μl, 0.225 mmol) and triethylamine (42 μL, 0.300 mmol) in MeCN (0.3 mL) and CH_2_Cl_2_ (0.15 mL), was added HBTU (114 mg, 0.300 mmol). After 1 h, the reaction mixture was diluted with AcOEt, and washed with 1N HCl, saturated NaHCO_3_ aq., and brine. The organic layer was dried over Na_2_SO_4_ and concentrated under reduced pressure. The residue was purified by silica gel chromatography (hexane/AcOEt = 1:1) to afford α-Sanshool (34.0 mg, 92%) as a colorless oil. ^1^H NMR (CDCl_3_) δ 0.89 (d, *J* = 6.4 Hz, 6H), 1.78-1.73 (m, 4H), 2.21-2.32 (m, 4H), 3.11 (t, *J* = 6.0 Hz, 2H), 5.33-5.37 (m, 1H), 5.55 (brs, 1H), 5.68 (dq, *J* = 14.4, 6.8 Hz, 1H), 5.77 (d, *J* = 15.2 Hz, 1H), 5.99 (dd, *J* = 11.2, 11.2 Hz, 1H), 6.04-6.18 (m, 2H), 6.30 (dd, *J* = 14.0, 11.2 Hz, 1H), 6.80 (dt, *J* = 15.6, 6.8 Hz, 1H). ^13^C NMR (CDCl_3_) 18.3, 20.1, 26.5, 28.6, 32.0, 46.8, 124.2, 125.2, 129.5, 129.6, 130.1, 131.7, 133.4, 143.4, 165.9. HRMS (ESI+) m/z calcd for C_16_H_25_NONa [M+Na]^+^: 270.1834, found: 270.1832.

**Methyl (2*E*,6*Z*,8*E*)-deca-2,6,8-trienoate (12)**

A mixture of **4d** (1.03 g, 2.00 mmol) in dry THF (10 mL) was cool to -78 °C under an argon atmosphere. KHMDS (4.4 mL, 2.20 mmol) was added dropwise to the solution over 2 min. After stirring at -78 °C for 0.5 h, crotonaldehyde (0.34 mL, 3.00 mmol) was added. After stirring for 2 h at -40 °C, the reaction mixture was quenched with saturated NH_4_Cl aq. and then extracted with AcOEt. The organic layer was dried with Na_2_SO_4_ and concentrated under reduced pressure. The residue was purified by silica gel chromatography (hexane/ AcOEt = 1:1) and (hexane/ AcOEt = 10:1) to give **12** (342 mg, 95%) as colorless oil. ^1^H NMR (CDCl_3_) δ 1.76 (d, *J* = 6.8 Hz, 3H), 2.26-2.32 (m, 4H), 3.71 (s, 3H), 5.20-5.26 (m, 1H), 5.71 (dq, *J* = 14.4, 6.8 Hz, 1H), 5.83 (d, *J* = 15.6 Hz, 1H), 5.96 (dd, *J* = 10.8, 10.8 Hz, 1H), 6.23-6.29 (m, 1H), 7.06 (dt, *J* = 15.6, 6.4 Hz, 1H). ^13^C NMR (CDCl_3_) δ 18.1, 26.0, 32.1, 51.2, 121.2, 126.5, 127.0, 129.0, 129.9, 148.4, 166.8. HRMS (ESI+) m/z calcd for C_11_H_17_O_2_Na [M+H]^+^: 181.1229, found: 181.1220.

**(2*E*,6*Z*,8*E*)-Deca-2,6,8-trienoic acid (13)**

A mixture of **12** (300 mg, 1.66 mmol) and NaOH (333 mg, 8.32 mmol) in water (3.3 mL) was stirred at 70 °C for 3 h. After cooling to room temperature, the reaction mixture was extracted with AcOEt, and then acidified with aqueous 1N HCl to pH = 2. The organic layer was washed with brine, dried over Na_2_SO_4_, filtered, and concentrated under reduced pressure to afford **13** (263 mg, 91%) as white solid. ^1^H NMR (CDCl_3_) δ ppm 1.76 (d, *J* = 6.8 Hz, 3H), 2.30-2.36 (m, 4H), 5.20-5.24 (m, 1H), 5.71 (dq, *J* = 14.4, 6.8 Hz, 1H), 5.84 (d, *J* = 16.0 Hz, 1H), 5.97 (dd, *J* = 10.8, 10.8 Hz, 1H), 6.23-6.30 (m, 1H), 7.06 (dt, *J* = 15.6, 6.4 Hz, 1H), 11.3 (brs, 1H). ^13^C NMR (CDCl_3_) 18.2, 25.9, 32.3, 121.1, 126.5, 126.9, 129.7, 130.1, 151.2, 172.1. HRMS (ESI+) m/z calcd for C_10_H_14_O_2_Na [M+Na]^+^: 189.0891, found: 189.0885.

**Spilanthol^4^**

To a mixture of **13** (106 mg, 0.638 mmol), isobutylamine (96 μl, 0.957 mmol) and triethylamine (0.18 mL, 1.23 mmol) in MeCN (1.3 mL) and CH_2_Cl_2_ (0.6 mL), was added HBTU (363 mg, 0.957 mmol). After 1 h, the reaction mixture was diluted with AcOEt, and washed with 1N HCl, saturated NaHCO_3_ aq. and brine. The organic layer was dried over Na_2_SO_4_ and concentrated under reduced pressure. The residue was purified by silica gel chromatography (hexane/AcOEt = 2:1) to afford spilanthol (116 mg, 84%) as a colorless oil. ^1^H NMR (CDCl_3_) δ 0.90 (d, *J* = 6.4 Hz, 6H), 1.74-1.76 (m, 4H), 2.23-2.31 (m, 4H), 3.13 (t, *J* = 6.4 Hz, 2H), 5.21-5.27 (m, 1H), 5.43 (brs, 1H), 5.68 (dq, *J* = 14.8, 6.8 Hz, 1H), 5.76 (d, *J* = 14.4 Hz, 1H), 5.95 (dd, *J* = 9.6, 9.6 Hz, 1H), 6.23-6.30 (m, 1H), 6.80 (dt, *J* = 15.6, 6.8 Hz, 1H). ^13^C NMR (CDCl_3_) 18.2, 20.0, 26.3, 28.5, 32.0, 46.8, 124.1, 126.6, 127.5, 129.3, 129.8, 143.3, 166.0. HRMS (ESI+) m/z calcd for C_14_H_23_NONa [M+Na]^+^: 244.1677, found: 244.1673.

1 Wu, B., Kun L., and Toy, P. H. (2012) Synthesis of hydroxy-α-sanshool. Synlett 23, 2564–2566. doi: 10.1055/s-0032-1317172

2 Lautens, M., Paquin, J.-F., Piguel, S., and Dahlmann, M. (2001). Palladium-catalyzed sequential alkylation-alkenylation reactions and their application to the synthesis of fused aromatic rings. J. Org. Chem. 66, 8127-8134. doi: 10.1021/jo0107296

3 Igarashi, Y. (2012). Alkynes and their use for preparation of sanshools in high stereoselectivity. Jpn. Kokai Tokkyo Koho Patent. P2012-116786A

4 Barbosa, A. F., de Carvalho, M. G., Smith, R. E., and Sabaa-Srur, A. U. O. (2016) Spilanthol: occurrence, extraction, chemistry and biological activities. Rev. Bras. Farmacogn. 26, 128–133. doi: 10.1016/j.bjp.2015.07.024

**3** ^1^H NMR

**7** ^1^H NMR

**4d** ^1^H NMR

**4d** ^13^C NMR

**8** ^1^H NMR

**8** ^13^C NMR

**10** ^1^H NMR

**1** ^1^H NMR

**1** ^13^C NMR

**11** ^1^H NMR

**11** ^13^C NMR

**12** ^1^H NMR

**12** ^13^C NMR

**13** ^1^H NMR

**13** ^13^C NMR

**Spilanthol** ^1^H NMR

**Spilanthol** ^13^C NMR

**
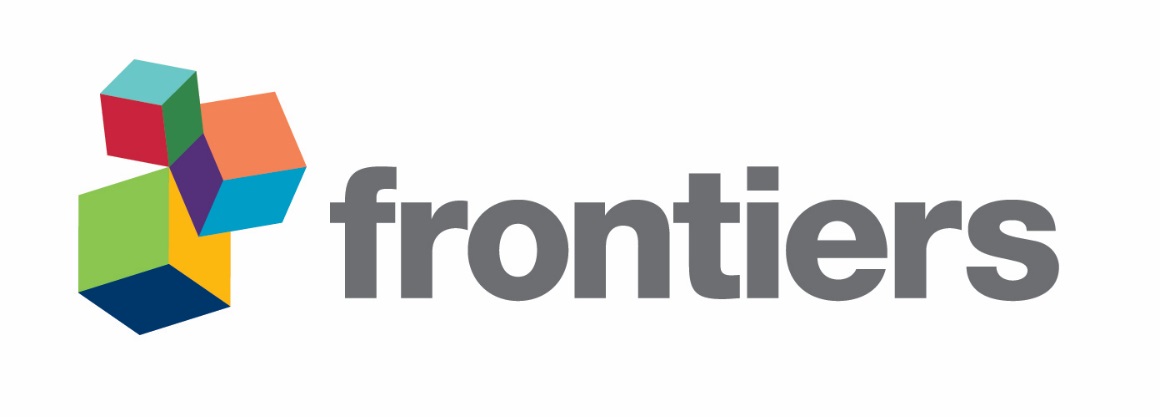
**
